# Screening, and Optimization of Fermentation Medium to Produce Secondary Metabolites from *Bacillus amyloliquefaciens,* for the Biocontrol of Early Leaf Spot Disease, and Growth Promoting Effects on Peanut (*Arachis hypogaea* L.)

**DOI:** 10.3390/jof8111223

**Published:** 2022-11-20

**Authors:** Taswar Ahsan, Chaoqun Zang, Shuyi Yu, Xue Pei, Jinhui Xie, Ying Lin, Xiaozhou Liu, Chunhao Liang

**Affiliations:** Institute of Plant Protection, Liaoning Academy of Agricultural Sciences, Shenyang 110161, China

**Keywords:** *Bacillus amyloliquefaciens* BAM, *Cercaspora ar* *achid* *ichola*, response surface method, central composite design, semolina flour

## Abstract

A novel *Bacillus amyloliquefaciens* BAM strain, with novel fermentation nutrient mediums and compositions, could produce potent antifungal secondary metabolites, as the existing strains face resistance from fungus pathogens. In the current study, we introduced two novel nutrient mediums for the fermentation process, semolina and peanut root extract, as carbon and nitrogen sources in order to maximize the antifungal effects of *B. amyloliquefaciens* against *Cercaspora arachidichola* to control early leaf spot disease in peanuts. Based on a single-factor test and the central composite design of response surface methodology, the optimum fermentation medium for *Bacillus amyloliquefaciens* antagonistic substance was determined, containing 15 gm/L of semolina flour, 12.5 gm/L of beef extract, and 0.5 gm/L of magnesium sulfate, which inhibited the fungal growth by 91%. In vitro, antagonistic activity showed that the fermentation broth of *B. amyloliquefaciens* BAM with the optimized medium formulation had an inhibition rate of (92.62 ± 2.07)% on the growth of *C. arachidichola*. Disease control effects in pot experiments show that the pre-infection spray of *B. amyloliquefaciens* BAM broth had significant efficiency of (92.00 ± 3.79)% in comparison to post-infection spray. *B. amyloliquefaciens* BAM broth significantly promoted peanut plant growth and physiological parameters and reduced the biotic stress of *C. archidechola*. Studies revealed that *B. amyloliquefaciens* BAM with a novel fermentation formulation could be an ideal biocontrol and biofertilizer agent and help in early disease management of early leaf spots in peanuts.

## 1. Introduction

Fungus pathogens usually attack the leaves of plants, and can adversely affect the entire plant [1]. Particularly in peanuts, the leaf is affected by fungus [2]. Early leaf spot disease in peanuts is caused by *Cercospora arachidicola* [3]. There is no doubt that *Cercosporoid* fungi are one of the most significant groups of plant pathogenic fungi that cause leaf spots. A wide range of plants (including cultivated plants on almost every continent) are affected by these diseases, including dicots, monocots, gymnosperms, and ferns [4,5]. To control fungus pathogens, fungicides have been proven to be the most effective; however, these chemicals cause environmental pollution and alarming human health concerns [6,7]. Global food security and sustainable agricultural productivity are two of the biggest challenges of the new millennium. To address these challenges, we need innovative food production technologies that minimize collateral damage to the environment and maintain the resilience of agroecosystems [8,9].

Therefore, agricultural products that are sustainable, efficient, and environmentally friendly have gained considerable attention in recent years [10]. There has been significant research on the effective control of plant diseases caused by various pathogenic fungi, bacteria, and viruses using biological control agents (BCAs), including Streptomyces, *Bacillus, Pseudomonas,* and *Trichoderma* microorganisms [11,12,13,14]. *Bacillus*-based agents are key components of biopesticides. Plant pathogens have been successfully controlled by several Bacillus species [15,16].

Their metabolites are recognized as effective plant growth promoters, biocontrol agents, mass producers, formulations, and commercially available compounds [17]. Growth promoters and the ability to suppress diseases on plants are the two main factors determining the value of microbial inoculants in agricultural applications [18,19]. Microbial synthesis to produce secondary metabolites is crucially dependent on the composition of the medium [20]. In microbial synthesis, the fermentation process is highly complex, which makes optimizing productive conditions an essential step. Physical parameters, as well as the composition of nutrient culture medium, can influence the microorganism fermentation processes [21]. The fermentation medium composition has a significant influence on the yield and metabolic profile of microorganisms [22]. Various fermentation parameters can be optimized by using classical and statistical tools. Fermentation optimization studies are often performed by the classical method, “one-factor-at-a-time (OFAT)” experiments, in which one factor is changed while the other remains constant [23].

There are a number of advantages to this method, including its simplicity and ease. However, the conventional OFAT method is time-consuming, inefficient, and labor-intensive. Furthermore, when several variables are involved, it does not take into account the combined effects of several nutritional and physiological parameters [24]. In order to achieve the optimization of a medium by changing more than one variable at a time, statistics-based measures have been implemented. Statistics-based experimental designs are often used in fermentation optimization, such as full factorial designs, fractional factorial designs, Plackett–Burman designs, Box–Behnken designs, and central composite designs [19,25].

In a multivariable system, response surface methodology (RSM) is used to design experiments and plot models, evaluate the impacts of various variables, and determine the optimum conditions for achieving significant results [26]. In addition to optimizing the factors of a fermentation medium, this method has been used extensively to discover the interaction between a multitude of fermentation parameters using minimal experiments [26]. To develop empirical models, RSM often uses statistics-based experimental designs such as BBD and CCD, which mathematically describe relationships between independent and dependent variables [27]. To clarify the shape of a response surface, RSM provides three-dimensional (3D) graphs and two-dimensional (2D) contour plots. The RSM method has been used successfully to optimize variables in fermentation cultures involving microorganisms such as fungi, bacteria, and actinomycetes that produce industrially important metabolites [28,29]. Biological control with Bacillus has been extensively researched in China and internationally. As of today, China has 110 registered plant diseases and insect pest control products (http://www.chinapesticide.org.cn/ accessed on 1 January 2021). In spite of this, there have been no reports of *Bacillus* species controlling peanut early leaf spots. The current study was carried out to screen and optimize the fermentation medium components to produce secondary metabolites from *Bacillus amyloliquefaciens* BAM. Furthermore, the disease control effects of *Bacillus amyloliquefaciens* BAM were evaluated against the *Cercospora archidechola* fungus for the disease management of early leaf spots in peanuts. Additionally, *Bacillus amyloliquefaciens* BAM was evaluated to study the growth-promoting effects on peanut plants.

## 2. Materials and Methods

### 2.1. Microorganisms

An antagonistic *Bacillus amyloliquefaciens* BAM (TL-6) strain was obtained from the lab of Liaoning Academy of Agricultural Sciences. The bacterial strain was maintained on LB medium at 4 °C. Pathogenic *Cercospora arachidichola* fungus was isolated from the infected peanut leaves and grown on a PDA medium.

#### Inoculum Suspension and Mode of Fermentation

The inoculum was prepared in 40 mL LB broth in a 250 mL flask. Two spore cakes (5 mm) from *B. amyloliquefaciens* BAM grown on LB synthetic medium agar were used as inoculants. We conducted submerged fermentation in a 250 mL Erlenmeyer flask with 45 mL of medium. Lysogeny broth medium (LB) consisted of 2.5 g/L sodium chloride, 10 g/L tryptone, and 7 g/L yeast extract. The pH was adjusted from 6.6 to 6.8 and sterilized at 121 °C, 15 lbs for 15 min. Later on, 5 mL of the suspension from the spores of *B. amyloliquefaciens* BAM was poured into each flask of fermentation medium. The medium was vigorously shaken at 30 °C for 96 h at 165 rpm. Upon completion of fermentation, the broth was assayed against *C. arachidichola*.

### 2.2. Screening of Key Factors in Fermentation Medium of BAM Strain by Single-Factor Experiment

The fermentation basal media were prepared following [30] with slight modifications. We used six mediums and LB as a control to screen the best medium for the production of secondary metabolites. Recipes are as follows: No. 1 (peptone 10.0 g/L, NH_4_Cl 5.0 g/L, MgSO_4_ 1.0 g/L, C_6_H_12_O_6_ 20.0 g/L, and soluble starch 20.0 g/L), No. 2 (yeast extract 3.0 g/L, sucrose 30.0 g/L, (NH_4_)_2_SO_4_ 10.0 g/L, KH_2_PO_4_ 0.3 g/L, and MgSO_4_ 5.0 g/L), No. 3 (semolina 16.0 g/L, corn flour 8.0 g/L, KH_2_PO_4_ 2.0 g/L, MgSO_4_ 0.5 g/L, and yeast extract 2.5 g/L), No. 4 (C_6_H_12_O_6_ 5.0 g/L, NaCl 10.0 g/L, yeast extract 2.0 g/L, and peanut root extract 3.0 g/L), No. 5 (C_6_H_12_O_6_ 10.0 g/L, KCl 5.0 g/L peptone 5.0 g/L, and yeast extract 8.0 g/L), No. 6 (MgSO_4_ 2.0 g/L, MnSO_4_ 0.04 g/L, corn flour 14.0 g/L, bean cake four 46.0 g/L, and corn steep liquor 3.0 g/L), and control LB medium (10 g/L tryptone, 5 g/L yeast extract, 5 g/L NaCl). All the different mediums contained different sole carbon and nitrogen sources. The fermentation broth was added to the PDA medium and then inoculated with *C. arachidichola* and incubated for 15 days, and the colony diameter of *C. archidechola* was measured to detect the antagonistic activity of the fermentation broth of the BAM strain. To screen the key nutritional factors for the fermentation of the BAM strain, carbon, nitrogen, and inorganic salts sources were as follows.

#### 2.2.1. Different Carbon Sources

An antagonistic activity test was conducted against *C. arachidicola* by replacing yeast extract with corn flour, semolina flour, soluble starch, sucrose, lactose, and peanut root extract in lysogeny broth medium. Lysogeny broth medium was used as a control.

#### 2.2.2. Different Nitrogen Sources

In order to test BAM fermentation filtrate’s antagonistic activity against *C. arachidichola*, beef extract, ammonia water, ammonium nitrate, ammonium sulfate, peptone, and peanut root extract were substituted for tryptone in LB medium, and LB medium was used as a control for the study.

#### 2.2.3. Different Inorganic Salts

In order to test BAM fermentation filtrate’s antagonistic activity against BAM, KH2PO_3_, MnSO_4_, MgSO_4_, FeSO_4_, CaCl_2_, and KCl were substituted for NaCl in LB medium, and LB medium was used as a control for the study.

#### 2.2.4. Experimental Design for Optimization of Nutrient Medium by Central Composites Design (CCD)

In the current study, a central composite design (CCD) of three factors–three levels was used with twenty base runs, demanding fifteen trials. The fractional factorial design comprises eight cube points, six center points, and six axial points with three parameters. To optimize fermentation broth for secondary metabolite production, the following parameters and levels were used: X_1_: semolina (0–15 mL%), X_2_: beef extract (0–15%), X_3_: MgSO_4_ (0–1.5%). The experimental central composite design is given in Table 1. The contour plots illustrate how independent variables interact with dependent variables. By using ridge maxima analysis and canonical analysis, it is possible to demonstrate the optimal mishmash of factors. By using MINITAB 22’s optimization function, this can be demonstrated. An optimal antifungal activity value was determined by the software response optimizer tool.

#### 2.2.5. Metabolic Profile in the Fermentation Batch

In the fermentation batch, dinitro salicylic acid was used to measure reducing sugars [31], and phenolic sulfuric acid was used to measure total sugars [32]. A ninhydrin reagent was used to determine amino nitrogen [33]. A pH meter was used to determine pH values of the fermentation batch at various intervals. Using the method of [34], dry cell weight was analyzed.

#### 2.2.6. Antagonistic Activity of BAM Strain against *C. arachidicola* In Vitro

The fermentation broth was used to determine the in vitro antagonistic activity of BAM against *C. arachidicola*. The treatments were designed as follows: optimized fermentation broth and basic fermentation broth; in control (Ck), a commercial fungicide was used. PDA was poured into a Petri dish and 200 μL of each treatment was added to it in the melted stage. Afterward, samples were inoculated with *C. arachidicola* and kept in a growth chamber at 28 °C for three weeks; later, we measured the inhibition growth. All the trials were performed in triplicate.

#### 2.2.7. Disease Control Effects of BAM Strain on Peanut Plants in Pot Experiments

To evaluate the disease control efficiency on peanut plants, pot experiments were conducted in the lab. The plants had full access to light, water, and air at room temperature. Seeds were sowed on the same day. After 20 days of growth, the peanut plants were given the treatments of fermentation broth from *Bacillus amyloliquefaciens* BAM, and for control, a commercial fungicide treatment was given. The treatment method was as follows. (1) Spray broth from *Bacillus amyloliquefaciens* (BAM) and then the next day inoculate with *Cercospora arachidicola* (CA). (2) Inoculate with CA and then the next day spray BAM. (3) Spray commercial fungicide (CF) and then the next day inoculate with CA. (4) Inoculate with CA and then the next day spray CF. After 14 days of the treatments, disease control effects were analyzed. Doses of 400 μL of both fungicide and BAM broth were sprayed onto each pot. The disease control efficiency was measured by counting the infection lesions on peanut leaves. All the trials were performed in triplicate.

#### 2.2.8. Determination of Growth and Physiological Parameters of Peanut Plants by the Effect of BAM Strain

To evaluate the growth and physiological parameters of peanut plants by the effect of BAM strain, pot experiments were conducted in the lab. The plants had full access to light, water, and air at room temperature. Seeds were sowed on the same day. After 20 days of growth, the peanut plants were given the treatments of fermentation broth from BAM. The treatment method was as follows. (1) Spray 200 μL + inoculate with *C. arachidicola* (CA). (2) Spray 400 μL BAM broth + inoculate with *C. arachidicola* (CA). (3) Spray 200 μL BAM broth. (4) Spray 400 μL BAM broth. (5) No treatment (Ck) and then inoculate with *C. arachidichola*. After 30 days of the treatments, growth effects and other physiological parameters were analyzed. In this experiment, we measured the photosynthetic rate (A), transpiration rate (E), stomatal conductance (gs), and sub-stomatal CO_2_ concentration (ci). All the experiments were performed in sunlight between 10 a.m. and 12 p.m. An open-system LCA-4 ADS was used to measure the gas exchange parameters (Analytical Development Company, Hoddesdon, England). All the trials were performed in triplicate.

#### 2.2.9. Statistical Analysis

A regression method of response surface was used to fit the experimental results of RSM. The equation is as follows:

Xi = Xi − Xi − /Xi = 1, 2, 3,…, K(1)

An independent variable’s coded value is Xi, its real value is X, its real mean is X, and its step change is Xi. Using a response curve fitting equation, we fitted the second-order polynomial model:

Y = b0 + bixi + i j bij xi xj + bii xi(2)

The response is measured by Y; the intercept term is indicated by b0; and bi, bij, and bii represent the effects of variables xi, xix, and xi, respectively. Xixj is a variable representing the interaction between xi and xj. To measure the goodness of fit of the regression, Fisher’s F-test, associated probability P(F), and determination coefficient R^2^ were analyzed by analysis of variance (ANOVA). In addition to the estimated coefficients and associated probabilities, P(t), Student’s *t*-values were included in the analysis. Surface plots were used to represent the quadratic models for each variable [35,36]. The statistical analysis (RSM) and regression analysis were performed using the statistical package Minitab 18. Graphs (surface plots) were drawn with Statistica 12. ANOVA analysis and Tukey tests were also performed with Statistica 12.

## 3. Results

### 3.1. Single-Factor Screening of Fermentation Medium for B. amyloliquefaciens Strain

**Figure 1:**
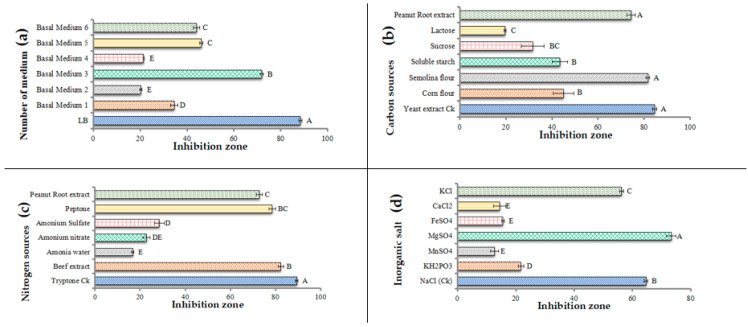
One factor, one time screening of fermentation medium of *B. amyloliquefaciens* BAM with different basal media (**a**), carbon sources (**b**), nitrogen sources (**c**), and inorganic salts (**d**). Means sharing similar letters in a column are statistically significant.

As shown in Figure 1a, the fermentation broth of the BAM strain cultured in the LB medium showed the highest inhibition rate of 88% against *C. arachidicola*. Thus, for further medium optimization, we selected LB medium with yeast extract (5 g/L) as the main carbon source, tryptone (10 g/L) as the main nitrogen source, and NaCl (10 g/L) as an inorganic salt. As different carbon sources were substituted for yeast extract in LB medium, the inhibition rate decreased in different ways (Figure 1b); among them, the fermentation filtrate of a BAM strain cultured with semolina flour as a carbon source exhibited the greatest inhibition rate (81%). The results show that semolina and peanut root extract were the best inhibitors, but they were not significantly different from yeast extract. The results of replacing tryptone in the fermentation basal medium with beef extract reveal that this substitute has a significant inhibitory effect of 82% against *arachidicola* (Figure 1c). Different inorganic salts were substituted for NaCl in the fermentation basal medium, and the inhibition rate of the fermentation filtrate of the BAM strain with magnesium sulfate as the inorganic salt was significantly greater than that of the fermentation filtrate of LB medium by 73% (Figure 1d). In the basic fermentation medium formulation, carbon, nitrogen, and inorganic salts were changed, and the production was combined with yeast extract, semolina flour, and peanut root extract as the best carbon sources; beef extract and peptone as the best nitrogen sources; and magnesium sulfate as the best inorganic salt. The roots of peanuts from BAM’s natural habitat replaced carbon and nitrogen sources, showing significant inhibition zones as well (Figure 1b,c).

### 3.2. Optimization of Fermentation Medium through CCD

A central composite design (CCD) based on response surface methodology was used to optimize the medium components in order to maximize the antifungal effects of secondary metabolites produced by BAM. A total of fifteen experiments were conducted using different concentrations of semolina flour, beef extract, and MgSO_4_. It was observed that the minimum antagonistic activity of secondary metabolites against *C. arachidicola* (41.51% inhibition zone) was observed in run number 11, while the maximum antagonistic activity (90.55%) was observed in run number 7 (Table 1). Second-order polynomial multiple regression was used to calculate the response obtained from experiments involving central composite designs. In Equation (3), Y is the response antifungal activity (Inhibition %), and X_1_, X_2_, and X_3_ are the coded values of the independent factors, namely semolina flour, beef extract, and MgSO_4_.

Y = −65.3 + 7.38X_1_ + 11.37X_2_ + 74.0X_3_ − 0.130X_1_ − 0.443X_2_^2^ − 21.2X_3_^2^ + 0.036X_1_^2^X_2_ − 3.50X_1_^2^X_3_ − 1.54X_2_^2^X_3_ (3)

Data collected from experiments were analyzed using analysis of variance (ANOVA), as shown in Table 2.

**Figure 2:**
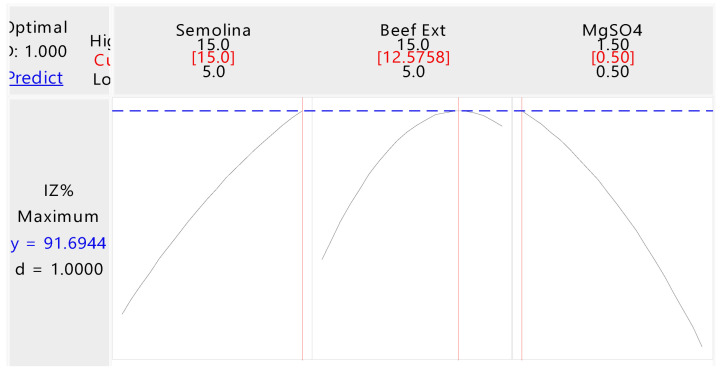
Predicted and actual values of media composition and inhibition zone against *C. arachidichola*.

Based on the Fischer test value of 10.59 and the p-value of 0.009, the proposed model was found to be significant. An adequate model coefficient of determination R^2^ demonstrated its appropriateness. The R^2^ values were also used to determine the trial parameters and their interactions, as well as the unpredictability of the response. According to the coefficient determination in this study, R^2^ = 0.9501 or 95.01% indicates that there was a variation of about 4.99% not explained by the model. This model was highly significant as well, as it had an adjusted determination coefficient R^2^ of 0.8604 or 86.04%. All the linear term regression coefficients showed a great impact on the secondary metabolites’ antagonistic activity according to the significance of corresponding p-values [pX_1_ = 0.006, pX_2_ = (0.014), and pX_3_ = (0.004)]. The antagonistic activity of the filtrate was greatly affected by semolina flour, beef extract, and magnesium sulfate. The quadric coefficients X_12_X_1_, X_22_X_2_, and X_32_X_3_ were significantly higher. These quadrants showed that increasing the parameter’s values amplified the activity of the antibiotic, and decreasing the parameter’s values decreased the activity of the antibiotic. Interactive term coefficients of X_1_^2^X_2_, X_1_^2^X_3_, and X_2_^2^X_3_ were significant, as shown in Table 3. Predicted and actual values of the medium and zone of inhibition against *C. arachidichola* are given in Figure 2.

**Figure 3:**
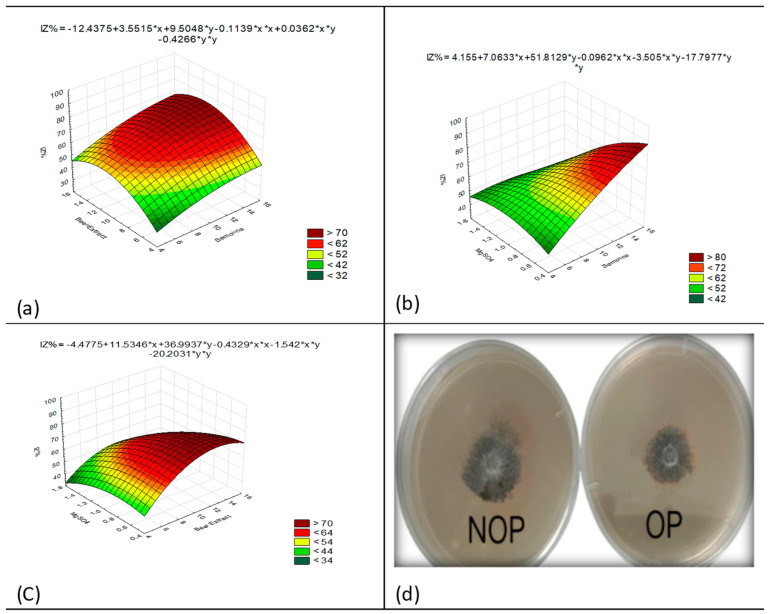
Response surface plots of the effect of various factors on the inhibition rate of *C. arachidichola* growth by fermented broth of BAM strain and validation of the optimal formulation. (**a**) The response surface plot for the effects of semolina flour and beef extract on inhibition rate at middle level of MgSO_4_ contents. (**b**) The response surface plot for the effects of semolina flour and MgSO_4_ on inhibition rate at middle level of beef extract contents. (**c**) The response surface plot for the effects of beef extract and MgSO_4_ on inhibition rate at middle level of semolina flour contents. (**d**) Validation of the optimal formulation for the inhibition rate of *C. arachidichola* growth by fermented broth of BAM. NOP (non-optimized), OP (optimized).

To determine the interactions between independent variables and the impact of each variable, regression models generated surface plots. Each surface plot of the independent variables indicates reciprocal interactions and signifies the signification of variables. Figure 3a–c shows the activity for each pair of variables keeping the others constant at their middle values. Figure 3a represents antagonistic activity as a function of semolina flour + beef extract by keeping MgSO_4_ fixed at an optimum value. The plot shows that lower and higher levels of semolina flour + beef extract support relatively low levels of secondary metabolite antagonistic activity, while the highest value of antagonistic activity of secondary metabolites obtained at the middle range of semolina flour + beef extract levels.

Figure 3b represents antagonistic activity as a function of semolina flour + MgSO_4_ by keeping beef extract fixed at an optimum value. The plot shows that lower and higher levels of semolina flour + MgSO_4_ support relatively low levels of secondary metabolite antagonistic activity, while the highest value of antagonistic activity of secondary metabolites was obtained in the middle range of semolina flour + MgSO_4_ levels. Figure 3c represents antagonistic activity as a function of MgSO_4_ + beef extract by keeping semolina fixed at an optimum value. The plot shows that lower and higher levels of MgSO_4_ + beef extract support relatively low levels of secondary metabolite antagonistic activity, while the highest value of antagonistic activity of secondary metabolites was obtained in the middle range of MgSO_4_ + beef extract levels. In Figure 3d, the inhibition of *C. archidechola* growth by the fermentation broth of BAM strain was increased by fermentation medium optimization.

### 3.3. Metabolism of BAM Strain in Submerged Fermentation

**Figure 4:**
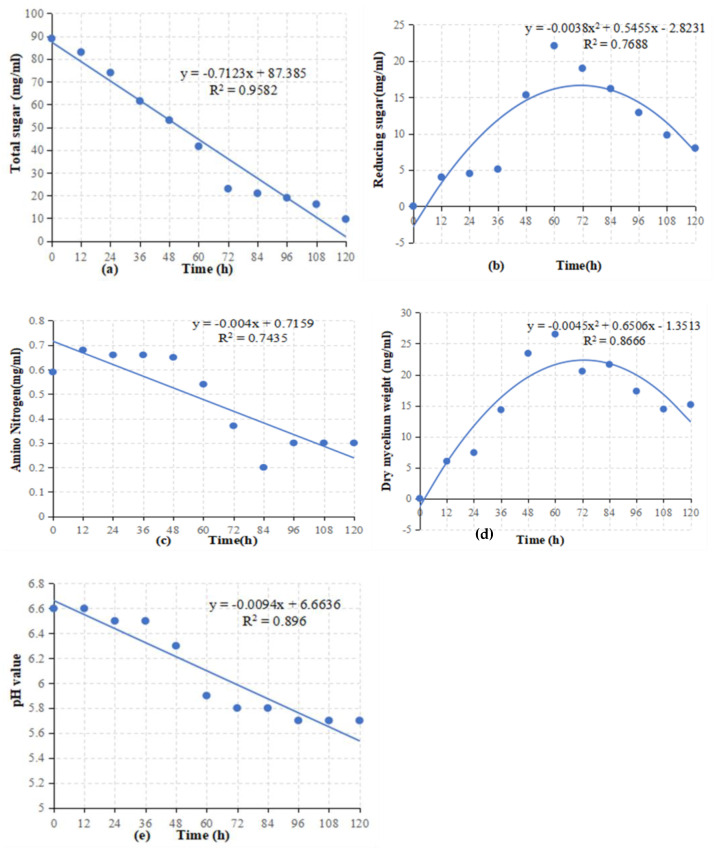
Metabolism study of submerged fermentation batch of BAM. Total sugar consumption (**a**), reducing sugar concentration (**b**), amino nitrogen (**c**), dry mycelium weight (**d**), pH values (**e**).

Total sugar, reducing sugar, amino nitrogen, pH, and dry mycelium weight were evaluated during fermentation. Exponential or linear models were used to explain the relationships between the independent variables. In the beginning, there was a high concentration of total sugar, but over time, the utilization of total sugar increased and the concentration decreased. A linear regression model describes the relationship between total sugar and time. As shown in Figure 4a, the exponential decay model provided the best-fitting regression (R^2^ of 0.9582). It started with low consumption, and as the total sugar was slowly utilized, the concentration of reducing sugar increased, and then abruptly decreased after 96 h. In a linear regression, reducing sugars are related to time. In this case, R^2^ was 0.7688. As shown in Figure 4b, there was an up-and-down trend in the relationship of amino nitrogen level with time described by linear regression. The value of R^2^ was 0.7435. Lines and equations indicate the best-fitting regression by the exponential decay model (Figure 4c). As fermentation progressed, the weight of dry mycelium increased. After 84 h, it reached a maximum, while after 120 h, the weight decreased. A linear regression model describes the relationship between dry mycelium weight and time. In this case, R^2^ was 0.866. An exponential decay model provides the best-fitting regression line (Figure 4d). In addition, the pH value in the fermentation process did not remain constant and fluctuated between 5.5 and 6.6. The relationship of pH with time was described by linear regression. In this case, R^2^ was 0.896. An exponential decay model provides the best-fitting regression line (Figure 4e).

### 3.4. Antagonistic Activity of BAM Strain against C. archidechola In Vitro

**Figure 5:**
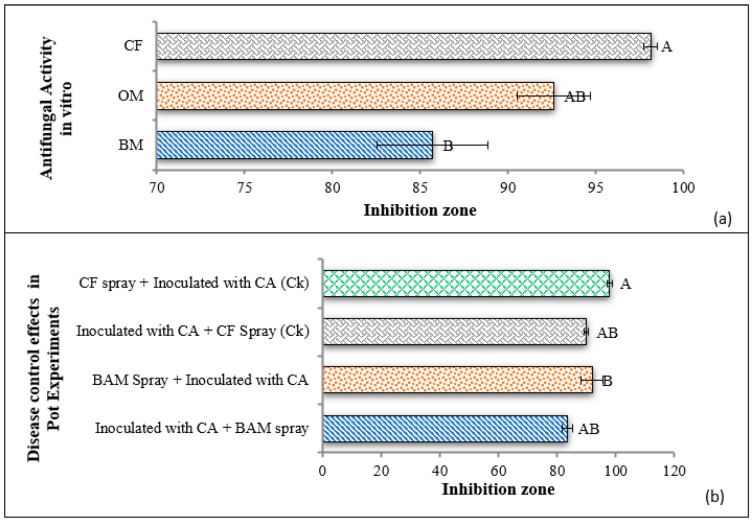
Antagonistic activity of BAM strain in vitro study. (**a**) CF (commercial fungicide) control was a control value. OM (optimized fermentation medium broth), BM (basic medium broth). (**b**) Disease control effects in pot experiments. CF = commercial fungicide, CA = *Cercospora arachidichola*.

The optimized fermentation broth exhibited significant antifungal activity of 92.62% against *C. arachidichola* in comparison to the basic medium fermentation broth, which showed antifungal activity of 85.73%. However, both optimized and basic mediums showed little antifungal activity compared to the control commercial fungicide. The control group exhibited 98.14% inhibitory antifungal activity, as shown in Figure 5a.

### 3.5. Disease Control Efficiency of BAM Strain in Pot Experiments

In pot experiments, disease control efficiency showed that BAM spray + inoculum of *C. arachidichola* had significant 92% control of early leaf spot disease; however, inoculum of *C. arachidichola* + BAM spray had less efficiency of 83.67% disease control. In the same way, in the control trials, CF spray + inoculum of *C. arachidichola* had strong and statistically significant disease control effects, while inoculum of *C. arachidichola* + CF spray both had less efficacy to control the early leaf spot (Figure 5b).

### 3.6. Effects of BAM Strain on the Growth and Physiology of Peanut Plants

**Figure 6:**
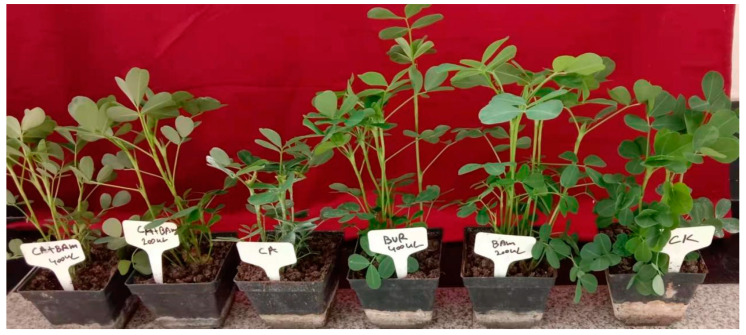
An illustration of the pot experiments on the growth of peanut plants by the effects of BAM strain. Ck (no treatment); BAM 200 μL and BAM 400 μL (treatment with fermentation broth of BAM strain); CA (inoculated with *Circospora arachidichola*); CA *+* BAM 200 μL and CA + BAM 400 μL (treatment with fermentation broth of BAM strain).

The BAM significantly promoted the growth of the peanut plants with biotic stress of *C. arachidichola* and without biotic stress. As the results showed, different doses of BAM fermentation broth increased the shoot and root length in comparison to control values. Treatment of 400 μL BAM had significant effects on weight of dry and fresh root and shoot. The values shown in the results are as follows: shoot fresh weight 21.00 ± 1.08a, shoot dry weight 11.00 ± 0.41a, root fresh weight 3.90 ± 0.08a, root dry weight 1.25 ± 0.13a. The results also showed that treatment of 400 μL BAM had significant effects on the length of shoot (16.75 ± 0.48 cm) and root (4.13 ± 0.38 cm). In the same way, the results of 400 μL BAM treated + inoculated with *C. arachidichola* showed increased fresh and dry weight: fresh shoot weight = 14.00 ± 0.71 g, shoot dry weight = 9.50 ± 0.29 g, root fresh weight = 1.98 ± 0.13 g, root dry weight 0.68 ± 0.03 g. The results also showed increased shoot (13.75 ± 0.25 cm) and root (3.12 ± 0.37 cm) length compared to the negative control (inoculated with *C. arachidichola*). The results are shown in Table 4. An illustration of the results is shown in Figure 6.

BAM strain significantly triggered the physiological parameters of peanut plants under the biotic stress of *C. arachidichola* and without biotic stress. The dose of BAM at 400 uL significantly influenced the photosynthesis rate, transpiration rate, stomatal conductance, and sub-stomatal CO_2_ concentration (163.78 ± 1.99 μmol CO_2_ m^−2^ s^−1^, 168.00 ± 5.45 mmol H_2_O^.^m^2^ s^−1^, 218.25 ± 7.09 H_2_O^.^m^−2^ s^−1^, and 202.43 ± 0.78 μmol CO_2_ mol^−1^, respectively). In the same way, the results of BAM treated + inoculated with *C. arachidichola* showed an increase in the parameters as compared to the negative control (inoculated with *C. arachidechola*). The results are shown in Table 5.

## 4. Discussion

In the current study, we screened and optimized the fermentation medium to produce secondary metabolites from a novel *Bacillus amyloliquefaciens* BAM strain to evaluate the biocontrol and PGR potential in order to control the *C. archidechola* to manage early leaf spot disease in peanut leaves. Zalila-Kolsi [19] stated that *Bacillus amyloliquefaciens* has a strong ability to control fungus pathogens and can trigger growth in different plants. However, fungus pathogens have developed resistance against several antagonists, as previously reported by [37,38]. Novel strains with novel formulations are thus urgently required to cope with this problem. In the current study, we introduced novel nutrient media for the fermentation process. Based on the antifungal activity against *C. arachidichola*, semolina and beef extracts were the significant optimized carbon and nitrogen sources, respectively, to produce secondary metabolites in the fermentation medium. Several studies revealed that the growth and bioactivity of microbes are greatly affected by the composition of the substrate medium, such as carbon and nitrogen sources and inorganic microelements [39,40]. A significant impact was observed on the antifungal activity of BAM with semolina flour as a carbon source in a submerged fermentation medium. In fermentation broth, semolina flour may promote BAM strain biomass production and its antifungal effects. According to studies, gluten and protein are found in greater amounts in semolina [41].

The results showed that the concentration of beef extract may be utilized to substitute tryptone extract as a nitrogen source in the fermentation of the BAM strain, which could also ensure the biocontrol impact of the BAM strain. In the submerged fermentation process of *Bacillus pumilus*, the beef extract has been described as an ideal source of nitrogen [42]. Additionally, NaCl in the basal medium can be effectively replaced with MgSO_4_ that has been optimized as an inorganic salt. Mg^2+^ has been shown to play a significant role in protein synthesis by taking part in the synthesis of amino acids, gene transcription and translation, the creation of proteins that serve as structural components of ribosomes, and the activation of a number of enzymes [42,43].

The results proved that the experimental design significantly affects the production of secondary metabolites to harbor the antagonistic activity of BAM against *C. arachidichola*. The results validated the performance of the central composite design of the response surface methodology. Response surface methodology is an appropriate mathematical and statistical tool for finding the best parameters to improve. It may also be useful to create an experimental design for revealing the relationships between various parameters. RSM has recently been utilized to improve fermentation parameters [44,45]. The appropriate medium content level and its interaction, together with the other three crucial factors, were determined by the results and were optimized using the CCD in RSM. Due to its efficiency and dependability, the central composites design is one of the best designs for fermentation [46,47]. Total sugar levels dropped in the fermentation batch as bacteria multiplied, but reducing sugar levels rose, showing that BAM was using both the total sugar and amino nitrogen to support its own growth. Later, a concentration of secondary metabolites and other metabolites in the fermentation batch together with a decrease in pH resulted in a slowdown in the BAM strain’s rate of growth. Utilizing complete sugar results in less reducing sugar being produced, which raises the degree of fermentation product synthesis while also having a positive impact on pH and a negative impact on the dry mycelium. Recently, [36,48] studied the same mechanism in *Streptomyces*.

In vitro antagonistic effects of BAM showed that optimized fermentation extract had strong biocontrol affects. BAM had sufficient antifungal effects against *C. arachidichola* to combat early leaf spot disease on peanut leaves. Semolina flour, beef extract, and ammonium sulfate were optimized ingredients for the high antifungal activity of secondary metabolites. Several studies indicate that optimized fermentation conditions enhance the antagonistic activity of *Bacillus* species against the fungus pathogens *Bacillus amyloliquefaciens* BLB369 [19] and *Bacillus pumilus* HR10 [42]. In pot experiments, disease control efficiency showed that BAM spray + inoculum of *C. arachidichola* had a significant effect on early leaf spot control on peanut plants; however, inoculum of *C. arachidichola* + BAM spray had less efficiency of disease control. This indicates that pre-treatment with BAM could control the disease more efficiently and enhance the immunity of the plant. This could be helpful for early disease management of early leaf spots and other *C. arachidichola*-related diseases. As stated by [49,50], endophytic bacteria, specifically *Bacillus* species, can control plant pathogens, as well as promote plant growth. Discovering and studying novel endophytic *Bacillus* species may provide new options in food and crop protection, specifically for developing microbial biocontrol agents that suppress post-harvest and pre-harvest fungal diseases [51]. Investigating the biological impact of BAM strain on *Arachis hypogea* growth, the results were observed in comparison to controls. The results demonstrated that the BAM fermentation broth has a significant impact on the growth of the plant. In comparison to the untreated control plants, treated plants had longer roots and shoots. Additionally, the fresh weight and dry weight of the treated plants were greater. When *Arachis hypogea* was treated with BAM, the fresh weight, dry weight, shoot length, and root length were significantly improved. Bacillus species, including *Bacillus subtilis*, *Bacillus amyloliquoefaciens*, *Bacillus cereus*, *Bacillus pumilus*, and *Bacillus polymyxa*, are widely recognized for their capacity for plant growth and development because they generate antibiotics as well as phytohormones [52]. The results showed that BAM broth treatment significantly affected the peanut plants’ physiological characteristics with and without the biotic stress of *C. archidechola*. The rate of photosynthesis was considerably impacted by the 400 µL dosage of BAM strain; it also had considerable impact on the rate of transpiration, stomatal conductance, and sub-stomatal CO_2_ concentration. Biomass was synthesized during photosynthesis from CO_2_ and H_2_O, which serves as the foundation for crop biomass and production. Ref. [53] demonstrated that some *Bacillus* species can activate certain physiological characteristics. Ref. [54] found that inoculating *Cicer arietinum* with *Bacillus subtilis* enhanced photosynthesis and pigment synthesis. Similarly, Ref. [55] reported that endophytic bacteria enhance growth, physiology, and metabolite profiles in plants. The results of their study emphasized that *Bacillus sp*. MN54 and *Bacillus phytofirmans* enhanced transpiration rate and stomatal conductance in marigolds (*Tagetes patula*). Our study demonstrated that *Bacillus amyloliquefaciens* BAM with novel fermentation nutrient medium composition could produce novel antifungal compounds. As there have been no reports of *Bacillus* species controlling peanut early leaf spots in China, according to our information, this was first study in this regard. We will continue this work to explore the functional gene responsible for producing metabolites and extract the pure compounds.

## 5. Conclusions

This study revealed that the novel source of nutrients in submerged fermentation can enhance antagonistic activity. Semolina as a carbon source influenced the production of secondary metabolites, which could help synthesize novel compounds to overcome pathogen resistance. The optimal concentration of novel carbon sources can enhance the activity of *Bacillus amyloliquefaciens* BAM. *Bacillus amyloliquefaciens* BAM broth had strong disease control efficiency and growth-promoting ability. This makes this product an ideal biocontrol and biofertilizer agent. Its pre-treatment spray will promote plant growth and resistance against early leaf spot disease in peanut. However, additional studies to explore novel compounds with different plant species should be conducted to draw further conclusions.

## Figures and Tables

**Table 1:** Experimental design and results of optimization of nutrient medium for the production of secondary metabolites from BAM strain by the central composite design.

StdOrder	RunOrder	PtType	Blocks	X_1_	X_2_	X_3_	Observed Values
5	1	2	1	−1 (5)	0 (10)	−1 (0.5)	55.61
9	2	2	1	0 (10)	−1 (5)	−1	53.90
2	3	2	1	1 (15)	−1	0 (1)	54.98
3	4	2	1	−1	1 (15)	0	56.45
7	5	2	1	−1	0	1 (1.5)	51.76
10	6	2	1	0	1	−1	71.54
6	7	2	1	1	0	−1	90.55
15	8	0	1	0	0	0	78.66
8	9	2	1	1	0	1	51.65
11	10	2	1	0	−1	1	45.32
1	11	2	1	−1	−1	0	41.51
13	12	0	1	0	0	0	68.54
4	13	2	1	1	1	0	73.54
12	14	2	1	0	1	1	47.54
14	15	0	1	0	0	0	65.65

Coded values: X_1_ = semolina, X_2_ = beef extract, X_3_ = MgSO4.

**Table 2:** Analysis of variance of antifungal activity against *C. arachidichola*.

Source	DF	Adj SS	Adj MS	F-Value	*p*-Value
Model	9	2512.13	279.126	10.59	0.009
Linear	3	1599.72	533.240	20.23	0.003
X_1_: Semolina	1	534.48	534.482	20.28	0.006
X_2_: Beef Extract	1	355.91	355.911	13.50	0.014
X_3_: MgSO_4_	1	709.33	709.326	26.91	0.004
Square	3	542.57	180.856	6.86	0.032
X_1_^2^X_1_	1	39.15	39.150	1.49	0.277
X_2_^2^X_2_	1	452.78	452.780	17.18	0.009
X_3_^2^X_3_	1	103.77	103.766	3.94	0.104
2-Way Interaction	3	369.85	123.282	4.68	0.065
X_1_^2^X_2_	1	3.28	3.276	0.12	0.739
X_1_^2^X_3_	1	307.13	307.126	11.65	0.019
X_2_^2^X_3_	1	59.44	59.444	2.25	0.193
Error	5	131.81	26.361		
Lack-of-Fit	3	38.46	12.821	0.27	0.842
Pure Error	2	93.34	46.671		
Total	14	2643.94			
Model Summary	S = 5.13432	R^2^ = 95.01%	R^2^ (adj) = 86.04%	R^2^ (pred) = 68.78%	

**Table 3:** Coded coefficients for antifungal activity against *C. archidechola*.

Term	Coef	SE Coef	T-Value	*p*-Value	VIF
Constant	70.95	2.96	23.93	0.000	
X_1_: Semolina	8.17	1.82	4.50	0.006	1.00
X^2^: Beef Extract	6.67	1.82	3.67	0.014	1.00
X_3_: MgSO_4_	−9.42	1.82	−5.19	0.004	1.00
X_1_^2^X_1_	−3.26	2.67	−1.22	0.277	1.01
X_2_^2^X_2_	−11.07	2.67	−4.14	0.009	1.01
X_3_^2^X_3_	−5.30	2.67	−1.98	0.104	1.01
X_1_^2^X_2_	0.91	2.57	0.35	0.739	1.00
X_1_^2^X_3_	−8.76	2.57	−3.41	0.019	1.00
X_2_^2^X_3_	−3.86	2.57	−1.50	0.193	1.00

**Table 4:** Effects of BAM strain on the growth of peanut plants; all the values are the comparison of mean ± SE.

**Treatment**	**Shoot Fresh Weight (g/plant)**	**Shoot Dry Weight (g/plant)**	**Root Fresh Weight (g/plant)**	**Root Dry Weight (g/plant)**	**Shoot Length (cm)**	**Root Length (cm)**
CF (Ck)	10.25 ± 0.48 ^c^	8.75 ± 0.25 ^ab^	3.08 ± 0.13 ^ab^	0.95 ± 0.08 ^ab^	15.00 ± 0.41 ^ab^	3.98 ± 0.23 ^ab^
200 μL BAM	20.00 ± 0.91 ^a^	10.00 ± 0.71 ^ab^	3.70 ± 0.14 ^b^	1.11 ± 0.06 ^b^	15.50 ± 0.65 ^a^	3.93 ± 0.25 ^ab^
400 μL BAM	21.00 ± 1.08 ^a^	11.00 ± 0.41^a^	3.90 ± 0.08 ^a^	1.25 ± 0.13 ^a^	16.75 ± 0.48 ^a^	4.13 ± 0.38 ^a^
Inoculated with CA	6.25 ± 0.25 ^d^	4.75 ± 0.63 ^c^	1.33 ± 0.23 ^c^	0.56 ± 0.07 ^c^	7.75 ± 0.48 ^d^	1.48 ± 0.18 ^c^
200 μL BAM + Inoculated with CA	11.75 ± 0.48 ^bc^	8.75 ± 0.25 ^b^	1.81 ± 0.20 ^c^	0.73 ± 0.11 ^bc^	12.50 ± 0.29 ^c^	2.73 ± 0.20 ^bc^
400 μL BAM + Inoculated with CA	14.00 ± 0.71 ^b^	9.50 ± 0.29 ^ab^	1.98 ± 0.13 ^c^	0.68 ± 0.03 ^bc^	13.75 ± 0.25 ^bc^	3.12 ± 0.37 ^c^

Means sharing similar letters in a column are statistically non-significant (*p* > 0.05). CA = *C. arachidichola*.

**Table 5:** Effects of BAM strain on the physiological parameters; all the values are the comparison of mean ± SE.

Treatment	Photosynthesis Rate (μmol CO_2_ m^−2^ s^−1^)	Transpiration Rate (mmol H_2_O m^2^ s^−1^)	Stomatal Conductance (mol H^2^O m^−2^ s^−1^)	Sub-Stomatal CO_2_ Concentration (μmol CO_2_ mol^−1^)
CF (Ck)	147.25 ± 2.43 ^ab^	149.25 ± 3.35 ^abc^	209.25 ± 4.85 ^a^	202.58 ± 0.55 ^a^
200 μL BAM	159.50 ± 2.96 ^a^	158.13 ± 5.14 ^ab^	169.75 ± 3.12 ^b^	202.75 ± 1.44 ^a^
400 μL BAM	163.78 ± 1.99 ^ab^	168.00 ± 5.45 ^a^	218.25 ± 7.09 ^a^	202.43 ± 0.78 ^a^
Inoculated with CA	120.03 ± 3.76 ^c^	116.50 ± 6.60 ^d^	138.00 ± 2.48 ^c^	202.78 ± 0.50 ^a^
200 μL BAM + Inoculated with CA	145.50 ± 4.84 ^ab^	135.00 ± 3.14 ^cd^	162.40 ± 3.86 ^b^	203.03 ± 0.26 ^a^
400 μL BAM + Inoculated with CA	149.25 ± 4.82 ^b^	143.00 ± 3.37 ^bc^	172.50 ± 6.44 ^b^	202.33 ± 0.72 ^a^

Means sharing similar letters in a column are statistically non-significant (*p* > 0.05).

## Data Availability

The data presented in this study are available on request from the corresponding authors.
